# Mef2c factors are required for early but not late addition of cardiomyocytes to the ventricle

**DOI:** 10.1016/j.ydbio.2020.11.008

**Published:** 2021-02

**Authors:** Duvaraka Kula-Alwar, Michael S. Marber, Simon M. Hughes, Yaniv Hinits

**Affiliations:** Randall Centre for Cell and Molecular Biophysics, School of Basic and Medical Biosciences and British Heart Foundation Centre of Research Excellence, Faculty of Life Sciences and Medicine, Guy’s Campus, King’s College London, London, SE1 1UL, UK

**Keywords:** Zebrafish, Second heart field, mef2c, Cardiomyocyte, Growth

## Abstract

During heart formation, the heart grows and undergoes dramatic morphogenesis to achieve efficient embryonic function. Both in fish and amniotes, much of the growth occurring after initial heart tube formation arises from second heart field (SHF)-derived progenitor cell addition to the arterial pole, allowing chamber formation. In zebrafish, this process has been extensively studied during embryonic life, but it is unclear how larval cardiac growth occurs beyond 3 days post-fertilisation (dpf). By quantifying zebrafish myocardial growth using live imaging of GFP-labelled myocardium we show that the heart grows extensively between 3 and 5 dpf. Using methods to assess cell division, cellular development timing assay and Kaede photoconversion, we demonstrate that proliferation, CM addition, and hypertrophy contribute to ventricle growth. Mechanistically, we show that reduction in Mef2c activity (*mef2ca*^*+/*^^−^*;mef2cb*^*−/−*^), downstream or in parallel with Nkx2.5 and upstream of Ltbp3, prevents some CM addition and differentiation, resulting in a significantly smaller ventricle by 3 dpf. After 3 dpf, however, CM addition in *mef2ca*^*+/*^^−^*;mef2cb*^*−/−*^ mutants recovers to a normal pace, and the heart size gap between mutants and their siblings diminishes into adulthood. Thus, as in mice, there is an early time window when SHF contribution to the myocardium is particularly sensitive to loss of Mef2c activity.

## Highlights

• Live image analysis showed extensive growth in zebrafish heart between 3 and 5 dpf.
• Proliferation, hypertrophy and addition of external CM progenitors contribute to heart growth.
• Reduced Mef2c activity, downstream or in parallel with Nkx2.5 and upstream of Ltbp3, diminishes growth resulting in a smaller ventricle by 3 dpf.
• SHF CM contribution to growth is particularly sensitive to loss of Mef2c at an early heart developmental time window.

## 1 Introduction

The heart is the first functional organ to form in amniotes (Buckingham et al., 2005). The early heart tube is formed of cells that are specified from lateral anterior splanchnic mesoderm and differentiate into cardiomyocytes (CMs) following combinatorial signals from neighbouring tissues (reviewed in (Kelly et al., 2014). Early heart development is well conserved amongst model organisms in key morphogenetic processes, specific genes involved and subsequent growth of the heart tube. Three modes of growth can be utilized in myocardial growth; addition of new CMs, proliferation of existing CMs and/or growth of CMs themselves (hypertrophy). Work in chick and mouse using dyes, transgenic lines, retrospective lineage analysis and genetic tracing with Cre recombinase has demonstrated that addition of cells to the arterial and venous poles of the heart tube occurs during heart growth (Cai et al., 2003; Kelly et al., 2001; Meilhac et al., 2003, 2004; Mjaatvedt et al., 2001; Verzi et al., 2005; Waldo et al., 2001; Zaffran et al., 2004). The progenitors of these late-added cells lie anterior to the heart in the pharyngeal region, referred to as the anterior or second heart field (SHF) to distinguish it from the first heart field, that forms the early or primitive heart tube. SHF cells are the main source of cells of the right ventricle and outflow tract (OFT) (reviewed in (Kelly et al., 2014). Addition of SHF-derived CM progenitors is an ongoing process that continues for a period of three days in the mouse, throughout the time of heart tube elongation and cardiac looping (Moorman et al., 2007; Prall et al., 2007) and in equivalent stages during chick development (Abu-Issa and Kirby, 2008; Tirosh-Finkel et al., 2006; van den Berg et al., 2009). Similarly to CM addition, hypertrophy of CMs also occurs at select locations in the chick heart. Older differentiated cells in the heart tube undergo hypertrophy and newly added CMs are smaller in size (Soufan et al., 2006). In mouse, hypertrophy has been shown to occur beyond heart looping stages by quantifying myofibril and cell length changes (Hirschy et al., 2006). Heart growth continues after the formation of heart chambers, and occurs at least partially from local proliferation (de Boer et al., 2012). However, it is not clear when the addition of cells to the heart from an external source stops, and how much each growth mode contributes at late developmental stages.

In zebrafish, atrial and ventricular precursor cells constituting the first heart field are localised at the marginal zone of the embryo at 5 ​h post-fertilisation (hpf), and following gastrulation migrate to form bilateral heart fields, which then migrate to the midline and fuse to form a cardiac disc. Simultaneously, endocardial cells have formed and become surrounded by the myocardial precursor cells. The disc everts into a cardiac cone, which remodels to give a two layered, beating heart tube by one day post fertilisation (dpf) (Bakkers, 2011; Stainier et al., 1993; Yelon, 2001). As in amniotes, in both fish and frog heart tube elongation is accompanied by addition of late differentiating SHF-like CMs to the OFT and inflow tract (IFT) (reviewed in (Knight and Yelon, 2016). Ventricular growth in the zebrafish prior to 3 dpf occurs by hypertrophy (Auman et al., 2007; Lin et al., 2012), CM addition (de Pater et al., 2009) and CM immigration (Staudt et al., 2014) with little CM division (de Pater et al., 2009). Most studies of CM development in zebrafish have concentrated on early development, tube formation, morphogenesis and looping in the first 2–3 days of development. Investigations of later heart growth in zebrafish have focused on the development of trabeculation. Trabeculae are sponge-like muscular structures extending into the cardiac cavity, increase muscle mass and are important for heart function. Trabeculae form between 2 and 3 dpf and increase in complexity in the days thereafter (Gupta and Poss, 2012; Liu et al., 2010; Peshkovsky et al., 2011; Rasouli and Stainier, 2017; Samsa et al., 2015; Staudt et al., 2014). Proliferation was shown to be essential for trabecular growth (Uribe et al., 2018). Whereas, some studies address growth occurring at later stages (Choi et al., 2013; Gupta and Poss, 2012; Hu et al., 2000; Shin et al., 2010), crucial quantitative and mechanistic understanding is lacking.

Gene networks that are involved in heart growth have been extensively characterised (reviewed in (Meilhac and Buckingham, 2018). Among the genes regulating SHF formation is the transcription factor Mef2C, a key regulator of sarcomeric myogenesis (reviewed by (Desjardins and Naya, 2016; Taylor and Hughes, 2017). In the mouse, *Mef2C* expression commences at E7.5 in the cardiogenic mesoderm (Dodou et al., 2004; Edmondson et al., 1994). *Mef2C* null mice die at 9.5 dpc with severe heart defects; the heart tube fail to undergo looping morphogenesis and only one hypoplastic ventricle is formed, made from a mixture of first heart field (FHF) and SHF progenitors (Bi et al., 1999; Lin et al., 1997, 1998; Materna et al., 2019; Verzi et al., 2005). In addition *Mef2C* null mice show severe vascular abnormalities and lack of smooth muscle differentiation (Bi et al., 1999; Lin et al., 1997, 1998). The mammalian *Mef2C* orthologues in zebrafish, *mef2ca* and *mef2cb*, express in the bilateral heart field and share overlapping functions driving CM differentiation. The double *mef2ca;mef2cb* mutant lacks most CMs and a functional heart, but has a residual differentiated CM population. *Mef2cb* is expressed strongly at the heart poles around 1 dpf and promotes addition of CMs to the OFT during the second dpf (Hinits et al., 2012; Lazic and Scott, 2011).

Here, we describe a method to quantify zebrafish myocardial growth during the trabeculation process. Using standardised methods to image live embryos with GFP-labelled myocardium and to quantify the ventricle in 3D, we show that the heart grows extensively between 3 and 5 dpf. CM addition, proliferation and hypertrophy contribute to this growth. Between 3 and 5 dpf a substantial contribution to heart growth is made by an *nkx2.5*-expressing cell population in the pharyngeal mesoderm anterior to the OFT. Mef2c expression is driven by Nkx2.5. Genetic reduction of Mef2c activity diminishes CM addition and differentiation resulting in a smaller ventricle by 3 dpf. However, after 3 dpf, CM addition in Mef2c mutants recovers to a normal pace, such that ongoing growth gradually reduces the myocardial difference between mutants and their siblings.

## 2 Materials and methods

### 2.1 Zebrafish lines, maintenance and manipulation

Zebrafish maintenance, staging and husbandry were as previously described (Westerfield, 2000). Mutant and transgenic lines: *Tg(myl7:EGFP)*^*twu26*^ (Huang et al., 2003), *Tg(myl7:EGFP-Hsa.HRAS)*^*s883*^ (D’Amico et al., 2007), *Tg(–5.1myl7:DsRed2-NLS)*^*f2*^ (Mably et al., 2003)*, Tg(myl7:GAL4FF)*^*hu6531*^(Strate et al., 2015), *Tg(5XUAS:RFP)*^*zf83*^ (Asakawa and Kawakami, 2008), *Tg(UAS:Kaede)*^*rk8*^ (Hatta et al., 2006), *mef2ca*^*b1086*^ (Miller et al., 2007) and *mef2cb*^*fh288*^ (Hinits et al., 2012) were maintained on AB wild-type background. *TgBAC(nkx2.5:GAL4FF)*^*hu9579*^ and *Tg(UAS:h2az2a-GFP)*^*hu6851*^ (Strate et al., 2015) lines were originally on TL wild type background.

Genotyping of *mef2ca*^*b1086*^ and *mef2cb*^*fh288*^ was performed by PCR and sequencing using the following primers 5′-ATTTCATGTCATGGAACTAAATCTGTT-3′ and 5′-AAGGCCAAACTCAACAGGAACT-3′ or 5′-CATTTGGCACCCTCTGTAAGT-3′ and 5′-ACTGAGCTGGAACTTACCTCC-3′ for b1086 allele, and for fh288 allele as described (Hinits et al., 2012).

### 2.2 In situ hybridisation and immunodetection

In situ mRNA hybridisation was performed as described previously (Hinits et al., 2009). Probes used were: *mef2ca* (Ticho et al., 1996), *mef2cb* (IMAGE:6519749) (Hinits et al., 2012), *mef2aa* (MGC:55208) (Thisse and Thisse, 2004), *myl7* (Yelon et al., 1999), *vmhc* (Yelon et al., 1999), *nkx2.5* (Lee et al., 1996) and *nppa* (Berdougo et al., 2003). Embryos were photographed as wholemounts on Leica dissecting microscope (MZ16/F) using Olympus DP70 camera. Immunodetection was performed as previously described (Hinits et al., 2012). Larvae older than 2 dpf were fixed with 2% paraformaldehyde in PBS for 30 ​min at room temperature. Antibodies used were against: Mef2 (c-21, Santa Cruz, 1:200), Mef2ca/cb (Anaspec, 1:200 (Hinits et al., 2012)), striated muscle myosin heavy chains (MyHC; A4.1025, 1:10 (Blagden et al., 1997), phospho-Histone H3 (PH3, Millipore 1:500), Nkx2.5 (GTX128357, GeneTex, 1:50) Elastin (Miao et al., 2007) 1:1000), GFP (rabbit, Torrey Pines 1:500 or chicken, Abcam ab13970, 1:500) and RFP (rabbit PM0005, Medical and Biological Laboratories, 1:500). Alexa dye-conjugated secondary antibodies (Invitrogen) were used at 1:1000. Hoechst 33342 (1:1000, Thermo Fisher Scientific) stained nuclei. Embryos/larvae were mounted in low melting point agarose and imaged on a Zeiss LSM Exciter confocal microscope using Zeiss 20×/1.0 NA dipping objective.

### 2.3 EdU treatment

Larvae at 4 dpf were incubated with 400 ​μM EdU in DMSO for 24 ​h in fish water. After treatment, larvae were rinsed, anaesthetized with tricaine and fixed in 4% PFA for 1 ​h. CLICK-IT-Alexa 594 (Invitrogen) was used to label EdU according to manufacturer’s protocol followed by immunodetection of GFP.

### 2.4 Imaging and data analysis

To measure heartbeat, embryos lightly anaesthetized with 0.61 ​mM tricaine were observed under a Zeiss stereo microscope (Stemi SV6) and heart rate was recorded at 1, 2 and 3 dpf. Fluorescent images were acquired on a Zeiss upright M1 stand LSM Exciter with a Zeiss 20×/1.0 ​W Plan Apochromat dipping objective using Zen software on larvae mounted in 1.5% low melting point agarose (LMA) in 30 ​mM butanedione 2-monoxime (BDM) to prevent cardiac contractions. The heart was imaged at gradually increasing intervals on scan settings optimised to compromise between bleaching and resolution for each embryo.

Images were processed on Volocity 6.0 software (PerkinElmer) prior to making measurements by re-slicing the image stack such that the maximal extent of ventricle and atrium lay in a single *XY* plane. The central *Z*-slice, defined as the longest length and widest width of the ventricle occurring in a single *Z* plane (generally halfway through the 3D stack), was used for thickness and axis measurements. Axial growth was measured from the apex to the OFT (*Y* axis), the width of the ventricle (*X* axis) from the outer curvature towards the atrioventricular junction and the depth of the ventricle (*Z* axis) orthogonal to the central *Z* slice to the most ventral point of the ventricle. Trabecular length was measured by manual tracing of the ‘skeletal length’ of the centre of the myocardial wall and trabecular projections and the ‘ventricular perimeter’ in the central *Z* slice and two additional slices orthogonal to the central *Z* slice and each other, and then subtracting the summed ventricular perimeter from the skeletal length. Ventricular wall thickness was measured from up to 8–12 randomised points in the central *Z* slice and two orthogonal axes by measuring the length of the GFP labelled myocardium from outer to inner portion on the ventricular wall. The ventral half of the ventricle had the highest image clarity and was utilized for volume and surface area measurements by thresholding for GFP. Embryos with DsRed^+^ nuclei and cytoplasmic GFP were used to quantify CM number. Nuclear counts were semi automated by thresholding for nuclear DsRed then manually separating connected nuclei. Graphs show means and standard error of the mean for the number of individual embryos shown. Statistical analysis was done with SPSS (*t*-test or ANOVA, with Bonferroni post-hoc tests where appropriate).

### 2.5 Mosaic labelling

For mosaic labelling, 1–2 ​cell *Tg(myl7:EGFP-Hsa.HRAS)*^*s883*^ embryos were injected with pCS2+_mCherry_kanR plasmid DNA (25 pg/embryo) (Bussmann and Schulte-Merker, 2011). Embryos were imaged live at 3 and 5 dpf.

### 2.6 Kaede lineage tracing

At 3 dpf, *Tg(myl7:GAL4FF)*^*hu6531*^*;Tg(UAS:Kaede)*^*rk8*^ embryos were mounted in 1.5% low melting point agarose and 30 ​mM BDM to stop the heart for the duration of imaging. Prior to photoconversion, hearts were imaged using a dual 488/543 excitation and a *Z*-stack collected. Immediately thereafter, a defined region in each embryo that included all heart Kaede-green-marked cells and their vicinity was photoconverted to Kaede-red by manual focusing throughout the *Z*-stack under continuous laser scanning using a 405 ​nm laser (Hatta et al., 2006) while assessing visually for residual green fluorescence. Embryos were re-scanned under dual excitation 488/543 ​nm post-photoconversion, then released from agarose and recovered in system water in the dark until 5 dpf when they were embedded and imaged again. Only larvae that were healthy after recovering were used for analysis. Photoconversion and imaging was undertaken on a Zeiss LSM Exciter confocal with a Zeiss W Plan-Apochromat 20×/1.0 DIC (UV) VIS-IR objective.

### 2.7 Adult fish measurements

Adult fish, reared in tanks together, were anaesthetized with tricaine, blotted dry, and weighed, standard length measured with a ruler nose-to-base of tail fin and fin-clipped for genotyping. Standard weight (K) was calculated using Fulton’s formula K ​= ​weight (g) x 100 × length^−3^ (cm) (reviewed in (Jones, 1999). A separate lay of fish were measured as above, followed by dissection of the heart. Hearts were imaged and ventricle length measured as described (Singleman and Holtzman, 2012).

## 3 Results

### 3.1 Extensive growth of the ventricle between 3 and 5 dpf

**Fig. 1:**
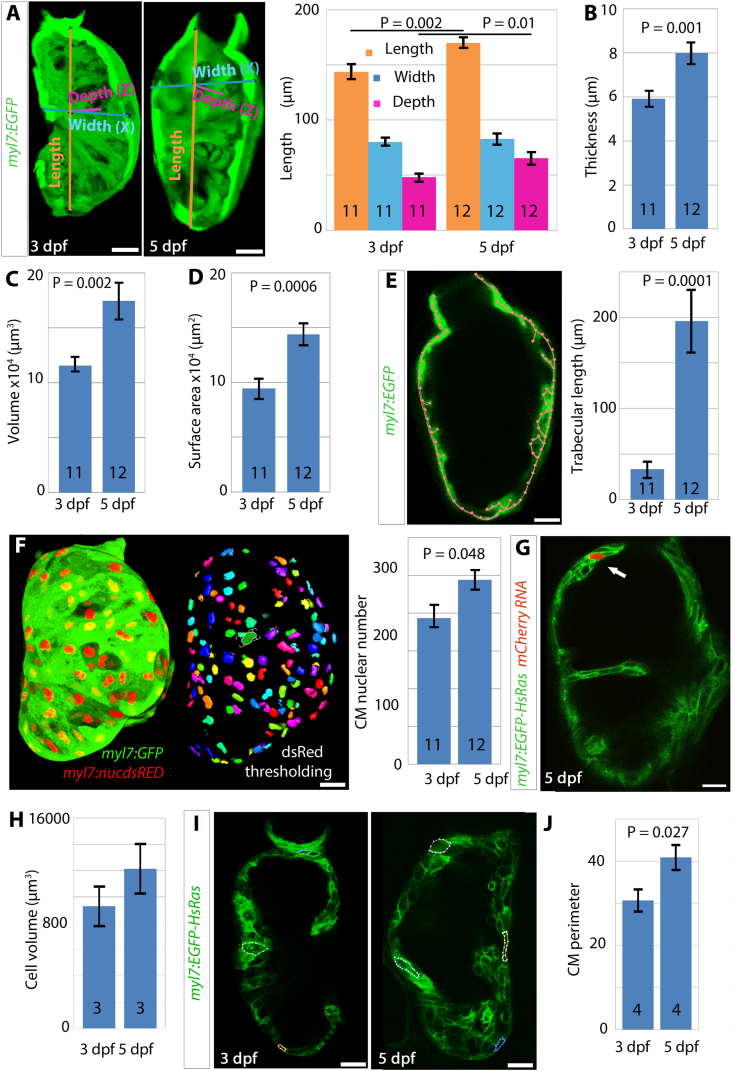
**Ventricular myocardium expands and thickens by CM addition. (A)** Ventricular axes were measured on confocal images of live *Tg(myl7:EGFP)* embryonic hearts at 3 and 5 dpf. Ventral half of ventricle shown. Graph shows increase in length of each axis. **(B)** Myocardial thickness increased significantly from 3 to 5 dpf. **(C,D)** Myocardial volume (C) and surface area (D) were measured by thresholding for GFP in scans of *Tg(myl7:EGFP)* embryos (see materials and methods), both increased significantly by 52% between 3 and 5 dpf. **(E)** Trabecular length increased 500% between 3 and 5 dpf. **(F)***Tg(myl7:EGFP);Tg(myl7:DsRed2-NLS)* were thresholded to count DsRed CM nuclei. Number of ventricular CMs increased by 26% from 3 to 5 dpf, n ​= ​5 and n ​= ​4 respectively. (**G,H**) Confocal section through *Tg(myl7:EGFP-Has.HRAS)*^*s883*^ scatter labelled with cytoplasmic mCherry (G, white arrow). Graph of average cell volume (H) measured from three hearts at 3 dpf (9 ​cells) or 5 dpf (10 ​cells). **(I,J)** Confocal optical sections through *Tg(myl7:EGFP-Has.HRAS)*^*s883*^ fish at 3 and 5 dpf with four CM perimeter measures carried out in each optical plane. Graph (J) shows cell measurements from four hearts for each time point. Values in all graphs are mean ​± ​SEM. N for each group shown on bars. Statistics used unpaired Student’s t-test. Scale bars ​= ​20 ​μm.

To quantify the growth of the zebrafish ventricle between 3 and 5 dpf, hearts of *Tg(myl7:egfp)*^*twu26*^ embryos were imaged live on a confocal microscope. Ventricular length, from the apex to the OFT, increased by 18% between 3 and 5 dpf, from 144 ​± ​6 ​μm to 170 ​± ​4 ​μm (p ​= ​0.002) (Fig. 1A). Thickness of the myocardial wall increased by 35% from 5.91 ​± ​0.32 ​μm ​at 3 dpf to 7.99 ​± ​0.46 ​μm ​at 5 dpf (p ​= ​0.001; Fig. 1B). To quantify the growth of the myocardium further, the GFP signal was thresholded and ventricular myocardial wall volume (referred to as ‘myocardial volume’ throughout the manuscript) and surface area measured using Volocity software (see Materials and Methods). Ventricular volume increased by 51% from 115,000 ​± ​6000 ​μm^3^ to 174,000 ​± ​15,000 ​μm^3^ from 3 to 5 dpf (p ​= ​0.002, Fig. 1C), whilst surface area increased by 57% from 94,000 ​± ​8000 ​μm^2^ to 144,000 ​± ​9200 ​μm^2^ (p ​= ​0.0006, Fig. 1D). To determine the contribution of trabeculation to this myocardial growth, we estimated trabecular length by subtracting perimeter from the skeletal length in the central slice of the ventricle (Fig. 1E). Trabeculae increased five-fold from 30 ​± ​8 ​μm to 196 ​± ​33 ​μm between 3 and 5 dpf (p ​= ​0.0001; Fig. 1E). Taken together, the data show that growth in ventricular myocardium size comprises increase in overall ventricle dimensions and in trabeculation. We next counted ventricular CM nuclei in *Tg(myl7:egfp)*^*twu26*^;*Tg(–5.1myl7:DsRed2-NLS)*^*f2*^ embryos and observed an increase of 26% between 3 and 5 dpf, from 193 ​± ​16 to 244 ​± ​11 nuclei (p ​= ​0.048; Fig. 1F). As CMs are close-packed and increase in number by 26% but myocardial volume increases by 51%, we calculated average volume per CM for each larva by dividing the volume by CM number from Fig. 1C and F. This revealed a 17% increase from 1200 ​μm^3^ ​at 3 dpf to 1400 ​μm^3^ ​at 5 dpf. We mosaically-labelled CMs by injecting the pCS2+_mCherry_kanR plasmid DNA, expressing cytoplasmic mCherry under CMV promoter, into *Tg(myl7:EGFP-Hsa.HRAS)*^*s883*^ embryos, in which myocardial cell membranes are labelled with EGFP (D’Amico et al., 2007) (Fig. 1G). At 3 dpf, average CM volume was 929 ​± ​152 ​μm^3^ (n ​= ​9) and rose to 1214 ​± ​190 ​μm^3^ (n ​= ​10) at 5 dpf, a 30% increase (Fig. 1H). Both methods suggest CM hypertrophy is occurring, although with some cell-to-cell heterogeneity. To assess hypertrophy using another independent method, we measured CM perimeter at 3 and 5 dpf from optical sections of larval hearts of *Tg(myl7:EGFP-Hsa.HRAS)*^*s883*^. We observed an increase of 41% in CM perimeter from 31 ​± ​15 ​μm ​at 3 dpf to 43 ​± ​17 ​μm (p ​= ​0.027; Fig. 1I and J). In conclusion, these various independent methods point to a contribution of CM hypertrophy to myocardial growth between 3 and 5 dpf. Taken together, the data show that ventricle growth results from increase in both CM number and size.

### 3.2 Cardiomyocyte increase between 3 and 5 dpf arises from both division and addition of progenitors from outside the heart

**Fig. 2:**
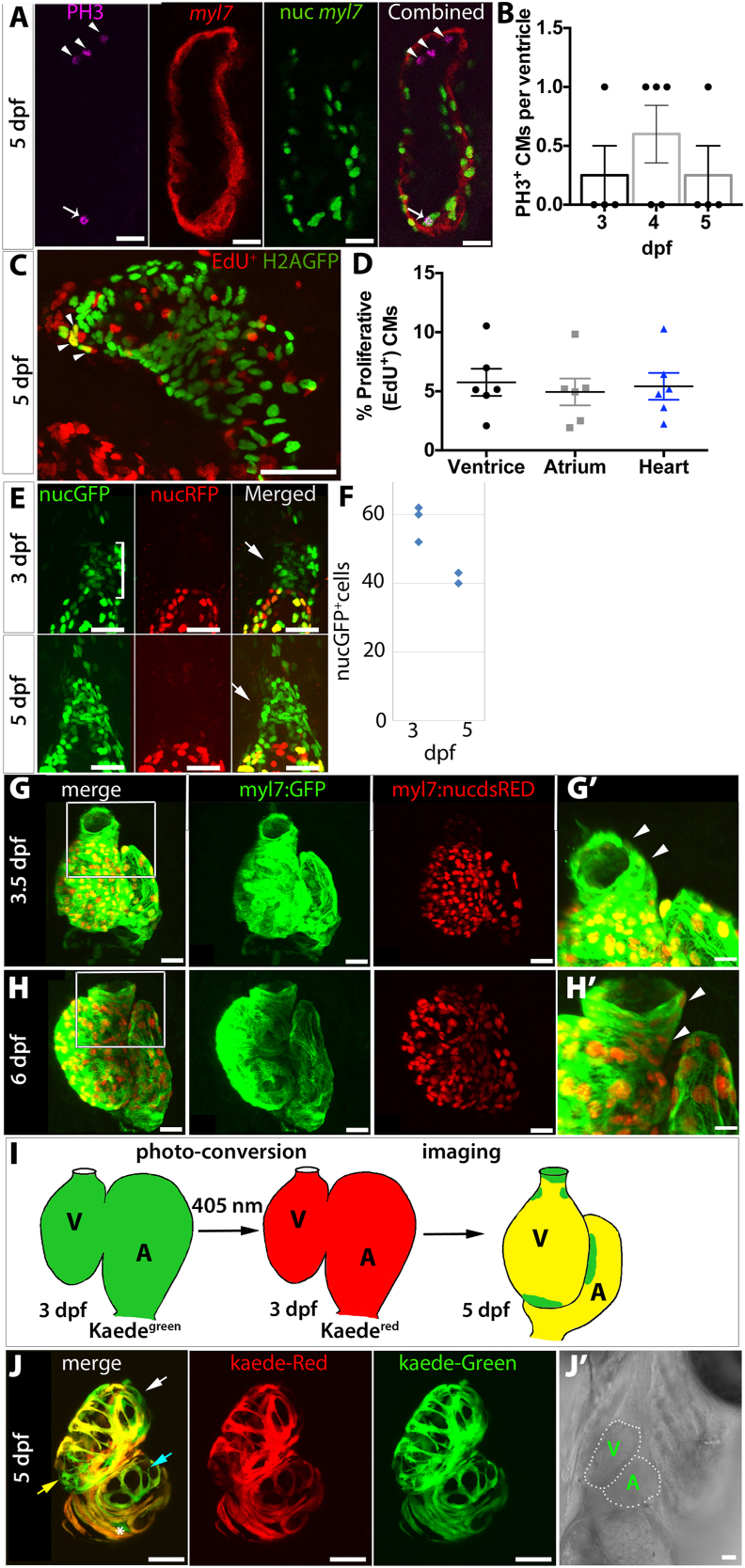
**CM addition, hypertrophy and proliferation are involved in ventricular growth between 3 and 5 dpf. (A)** Three Optical sections of 5 dpf *Tg(myl7:GAL4FF)*^*hu6531*^*;Tg(5XUAS:RFP)*^*zf83*^*;Tg(UAS:h2az2a-GFP)*^*hu6851*^ larval heart immunostained for GFP (green), RFP (red) and PH3 (magenta) to reveal the sole PH3^+^nucGFP^+^ CM detected in this heart (arrow). Note the additional PH3^+^nucGFP^−^ cells enclosed by the myocardial layer (arrowheads). **(B)** Number of PH3^+^ CMs/ventricle at 3 dpf, 4 dpf and 5 dpf. Mean ​± ​SEM. Each dot represents an animal. **(C,D)***Tg(myl7:GAL4FF)*^*hu6531*^*;Tg(UAS:h2az2a-GFP)*^*hu6851*^ embryos were exposed to EdU between 4 and 5 dpf. A short confocal stack reveals EdU-labelled CMs (arrowheads, C), which were quantified as the fraction of EdU^+^ nuclei in H2A-GFP-labelled CMs in ventricle, atrium and whole heart (D). **(E,F)** Confocal images of anterior ventricle and OFT region of a Tg*(nkx2.5:galFF);Tg(UAS:h2a-gfp);Tg(myl7:nucDsRed)* larva imaged at 3 and 5 dpf showing a nucGFP^+^;nucDsRed^−^ cell population (white bracket) adjacent to the OFT region (arrow) at each stage (E). nucGFP^+^ cells were counted in the bracketed areas (F). **(G,H)** Confocal stacks of embryos of 3.5 (G) and 6 dpf (H) carrying *Tg(myl7:EGFP)*^*twu26*^ and *Tg(–5.1myl7:DsRed2-NLS)*^*f2*^. G′ and H′ show in greater detail the boxed OFT region. EGFP^+^ dsRed^−^ cells are clearly present at 3.5 dpf indicating recently differentiated CMs (arrowheads), whereas by 6 dpf all cells seem to be positive for both EGFP and DsRed. **(I)** Schematic diagram of the Kaede conversion experiment. **(J)** Confocal stacks of hearts 5 dpf embryos carrying *Tg(myl7:GAL4FF)*^*hu6531*^*and Tg(UAS:Kaede)*^*rk8*^ in which Kaede was converted from green to red at 3 dpf. Transmitted light of the scanned area is shown in J’. Cells expressing green Kaede but no or little red Kaede are marked by white arrow (OFT and ventricle nearby), light blue arrow (AVC), and yellow arrow (ventricle apex). Note, atrial cell expressing green Kaede only (asterisk) was found only in this embryo. Scale bars ​= ​50 ​μm, except A and E ​= ​20 ​μm.

CM number could increase either due to CM division or addition of cells from an external source. To assess CM proliferation, embryos with myocardium positive for both nuclear GFP and cytoplasmic RFP expression, *Tg(myl7:GAL4FF)*^*hu6531*^*;Tg(5XUAS:RFP)*^*zf83*^*;Tg(UAS:h2az2a-GFP)*^*hu6851*^, were fixed at 3, 4 or 5 dpf and immunostained for the M-phase cell cycle marker phospho-Histone H3 (PH3, Fig. 2A) (Wang and Higgins, 2013). We found little evidence for proliferation in the ventricle at these stages with this method; no ventricles were observed with more than one PH3^+^ CM and several ventricles had no PH3^+^ CMs (Fig. 2B), similar to a previous study (Uribe et al., 2018). We therefore used incorporation of 5-ethynyl-2′-deoxyuridine (EdU) to detect DNA synthesis during S phase (Fig. 2C) (Salic and Mitchison, 2008). We incubated *Tg(myl7:GAL4FF)*^*hu6531*^*;Tg(UAS:h2az2a-GFP)*^*hu6851*^ embryos with EdU for 24 ​h from 4 dpf to 5 dpf and found that around 5% of ventricular CMs were EdU^+^ (Fig. 2D). Similar proliferation levels were also found in atrial CMs (Fig. 2D). Thus, proliferation contributes to larval CM increase, as previously reported (Uribe et al., 2018), but appears insufficient to explain the 26% increase between 3 and 5 dpf.

Another explanation for increase in CM number is addition of CMs from an external source, as occurs before 2 dpf (de Pater et al., 2009; Hami et al., 2011; Lazic and Scott, 2011; Zhou et al., 2011), and was shown to derive primarily from Nkx2.5^+^ progenitor population (Colombo et al., 2018; Guner-Ataman et al., 2013; Paffett-Lugassy et al., 2017; Zhou et al., 2011). When *TgBAC(nkx2.5:GAL4FF)*^*hu9579*^*;Tg(UAS:h2az2a-GFP)*^*hu6851*^ fish were crossed to *Tg(–5.1myl7:DsRed2-NLS)*^*f2*^, some nuclei in the OFT at 3 and 5 dpf were labelled with both markers (GFP^+^;DsRed^+^), indicating that they are differentiated CMs. In addition, a population of GFP^+^;DsRed^−^ nuclei, expressing *nkx2.5* but not *myl7* were observed just anterior to the OFT region and numbered 50–60 ​cells at 3 dpf and ~40 ​cells at 5 dpf (Fig. 2E and F). These GFP^+^;DsRed^−^ *nkx2.5*-expressing cells were positioned where, at earlier times, cells were shown to contribute to myocardial and non-myocardial cells in the OFT, as well as to pharyngeal cells (Colombo et al., 2018; Guner-Ataman et al., 2013; Paffett-Lugassy et al., 2017; Zhou et al., 2011). These data suggest that *nkx2.5*-expressing cells anterior to the heart provide an external source of CM increase between 3 and 5 dpf.

To test whether CMs are added to the OFT from the pharyngeal region after 3 dpf, we used a developmental timing assay shown to work at earlier stages of development (de Pater et al., 2009) employing embryos carrying *myl7*-driven transgenes expressing both EGFP and nucDsRed (de Pater et al., 2009). As fluorescence of nucDsRed protein takes approximately 24 ​h longer than EGFP to mature, recently-formed CMs are EGFP^+^nucDsRed^−^, whereas older CMs are EGFP^+^nucDsRed^+^. At 3.5 dpf, many CMs in the OFT were EGFP^+^nucDsRed^−^ (Fig. 2G-G′) indicating recent addition. By 6 dpf, all CMs in the OFT region were EGFP^+^nucDsRed^+^ (Fig. 2H, H′). These findings suggest that addition of CMs to the OFT had ceased prior to ~5 dpf. To test this idea further, photoconversion of the fluorescent protein Kaede from green to red (Ando et al., 2002) was employed to examine the timing of CM addition to the heart. Green Kaede cells in the hearts of *Tg(myl7:GAL4FF)*^*hu6531*^;*Tg(UAS:kaede)*^*rk8*^ larvae were converted to red Kaede using 405 ​nm laser at 3 dpf (Fig. 2I and S1). Subsequently, at 5 dpf, 4/4 converted embryos had re-appearance of cells expressing only green Kaede at the arterial pole and in the adjacent anterior ventricle (Fig. 2I,J, J’). Some CMs expressing predominantly green Kaede were also observed at the apex of the ventricle and near the atrioventricular canal (AVC) (Fig. 2G and H). The combined data show that addition of CMs from outside the arterial pole continues beyond 3 dpf, contributing to the growth of the ventricle and OFT together with proliferation and hypertrophy of existing CMs.

### 3.3 Mef2cb is required for normal ventricle size

**Fig. 3:**
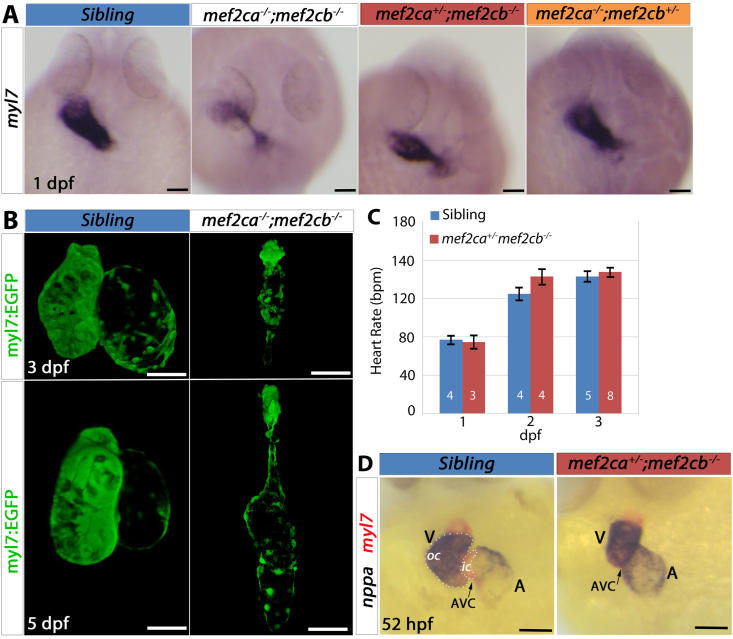
**Mef2c is required for the formation of the heart. (A)** In situ mRNA hybridisation for *myl7* on 1 dpf embryos from an incross of *mef2ca*^+/^^−^;*mef2cb*^+/^^−^ in dorsal view, anterior to top, sequence genotyped retrospectively. Images are representative of the majority of embryos of each genotype. *mef2ca*^−/^^−^;*mef2cb*^−/^^−^ embryos had a severe reduction in *myl7* expression. *mef2ca*^+/^^−^;*mef2cb*^−/^^−^ also showed a reduction in *myl7* expression but to a lesser extent than double homozygous mutants. **(B)** Confocal stack of live hearts of *mef2ca*^*−/−*^*;mef2cb*^*−/−*^ and its sibling on a *Tg(myl7:EGFP)* background. Double homozygous mutants continue to show a severe heart defect at 3 and 5 dpf but some growth in the differentiated structure is observed. **(C)** Heart rate measurements at 1–3 dpf for genotyped embryos showing no differences in heart rate of *mef2ca*^*+/*^^−^*;mef2cb*^*−/−*^ embryos compared with their siblings (unpaired Student’s t-test, P ​> ​0.05). Values are mean ​± ​SEM. N for each group shown on bars. (**D)** Hearts of 52 hpf embryos from a cross of *mef2ca*^+/^^−^;*mef2cb*^+/^^−^ to *mef2cb*^*−/−*^ shown in ventral view after a double in situ hybridisation for *nppa* (blue) and *myl7* (red). Sibling shown is *mef2ca*^*+/+*^*;mef2cb*^*+/*^^−^. Hearts of *mef2ca*^*+/*^^−^*;mef2cb*^*−/−*^ are smaller and underdeveloped. Scale bars ​= ​50 ​μm.

We have previously shown that lack of Mef2ca and Mef2cb, the zebrafish orthologues of mammalian Mef2C, results in severe lack of CM differentiation and the formation of an abnormal string-like non-functional heart at 2 dpf (Hinits et al., 2012). In situ mRNA hybridisation (ISH) of embryos from incrosses of *mef2ca*^*+/*^^−^*;mef2cb*^*+/*^^−^ fish showed severe reduction of myocardial differentiation markers in the double homozygous mutants. Between 22 somite stage (22 ​s ​s) and 24 hpf, *myl7* expression was grossly downregulated in the fraction (6.25%) predicted for double mutants (6/85 ​= ​7%) (Fig. 3A and S2A). Sequencing of genomic DNA from these embryos showed that 5/5 embryos with a string-like heart were indeed *mef2ca*^*−/−*^*;mef2cb*^*−/−*^ mutant embryos. Similar results were found for CM markers *bmp4* (5/88 ​= ​5.7%) and *vmhc/myl7* (3/86 ​= ​3.5%) (Figs. S2A–C). Samples of each were confirmed as *mef2ca*^*−/−*^*;mef2cb*^*−/−*^ mutants by sequencing.

To test whether Mef2c genes continue to control CM addition occurring between 3 and 5 dpf, we examined embryos from the dual heterozygote incross at later stages. *mef2ca*^*−/−*^*;mef2cb*^*−/−*^ double mutants had a string-like beating heart, which appeared to grow between 3 and 5 dpf (Fig. 3B). However, double mutant embryos did not survive beyond 7–10 dpf. In addition to the severe double mutant phenotype, a further fraction of embryos from the dual heterozygote incrosses had milder but significant defects in heart tube size at the linear heart tube stage: 15/85 (17.6%, *myl7*), 19/88 (21.6%, *bmp4*) and 25/86 (29%*, vmhc/myl7*) (Fig. 3A and S2A-C). Most, but not all (4/5), *mef2ca*^*+/*^^−^*;mef2cb*^*−/−*^ embryos had hearts with clearly reduced *myl7* mRNA and an abnormal shape (Fig. 3A). Some *mef2ca*^*−/−*^*;mef2cb*^*+/*^^−^ embryos (2/4) were also found to have less signal (data not shown), but others did not (2/4, Fig. 3A). Other genotyped embryos with normal size heart tubes were all from other genotypes with at least two wild type alleles of *mef2ca* and/or *mef2cb* (10/10, Fig. 3A and data not shown). Thus, having only a single wild type allele of either *mef2ca* or *mef2cb* was insufficient to prevent reduction in heart size at heart tube stage.

All *mef2ca* homozygous mutant embryos develop a jaw phenotype and die from lack of feeding (Hinits and Hughes, 2007; Miller et al., 2007; Nichols et al., 2016), whereas *mef2ca*^*+/*^^−^*;mef2cb*^*−/−*^ fish survive without any apparent jaw defects, allowing further study of heart growth and function. Despite their frequently abnormal size and shape, hearts of *mef2ca*^*+/*^^−^*;mef2cb*^*−/−*^ had no difference in heart rate compared with their siblings between 1 and 3 dpf (Fig. 3C). By 52 hpf, the heart tube had grown and underwent morphogenesis into the expanded chambers stage in which the ventricle is kidney shaped, with distinctive outer and inner curvatures (OC and IC) (Auman et al., 2007). At this stage, *nppa* mRNA accumulates in the OC, but is absent from the IC and the atrioventricular canal (AVC; Fig. 3D). ISH for *nppa* on embryos from a cross of *mef2ca*^*+/*^^−^*;mef2cb*^*+/*^^−^ with *mef2cb*^*−/−*^ showed that *mef2ca*^*+/*^^−^*;mef2cb*^*−/−*^ embryos had a small, rounded and misshapen ventricle without the typical unstained gap between the atrium and the ventricle, where the IC and the AVC lie (4/5, Fig. 3D). All other genotypes showed normal heart shape and *nppa* mRNA (14/14, Fig. 3D and data not shown). Thus, *mef2ca*^*+/*^^−^*;mef2cb*^*−/−*^ mutant ventricles show a morphological defect at the chamber expansion stage.

**Fig. 4:**
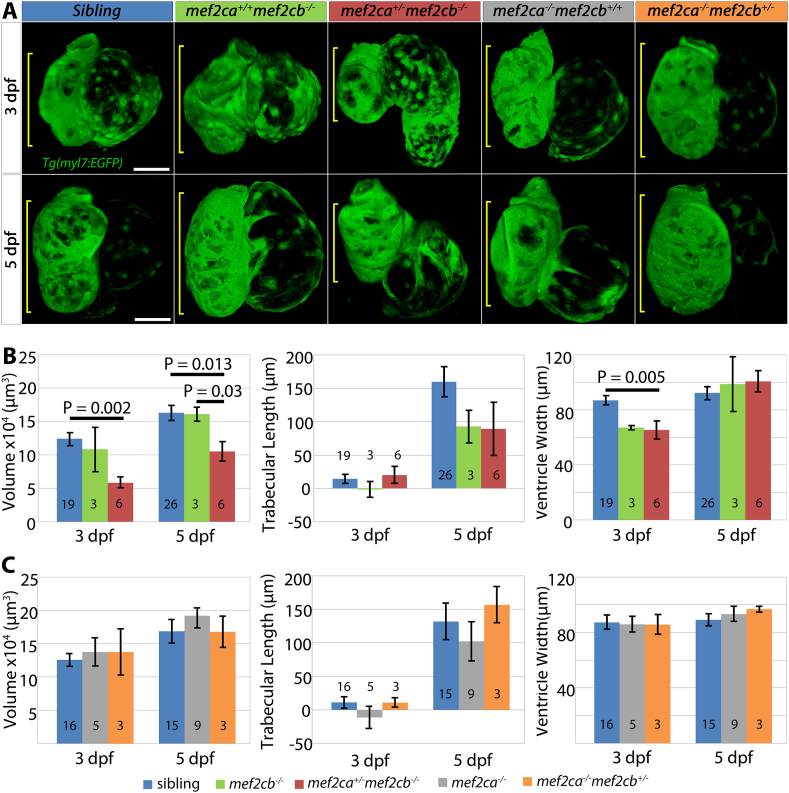
***Mef2ca***^***+/***^^−^***;mef2cb***^***−/−***^**mutant embryos show reduced ventricular size compared to siblings. (A)** Confocal images of 3 dpf (top panels) or 5 dpf hearts (bottom panels) from a cross of *mef2ca*^*+/*^^−^*;mef2cb*^*+/*^^−^*;Tg(myl7:EGFP)* to either *mef2ca*^*+/+*^*;mef2cb*^*+/*^^−^*;Tg(myl7:EGFP)* or *mef2ca*^*+/*^^−^*;mef2cb*^*+/+*^*;Tg(myl7:EGFP)* fish. *mef2ca*^*+/*^^−^*;mef2cb*^−/^^−^ embryos have a smaller heart at 3 and 5 dpf. Scale bars ​= ​50 ​μm. **(B,C)** Volume, trabecular length and width were quantified for embryos from crosses in A designed to test Mef2ca (B) and Mef2cb (C) function. One-way ANOVA analysis followed by Bonferroni post hoc tests, p-values indicated on graphs. Graphs show mean ​± ​S.E.M. N for each group shown on bars.

To quantify changes in ventricular size of larvae with reduced Mef2c, we analysed cardiac growth parameters at 3 and 5 dpf for all genotypes carrying at least one wild type allele of *mef2ca* or *mef2cb* (Fig. 4). At 3 dpf, *mef2ca*^*+/*^^−^*;mef2cb*^*−/−*^ mutants had significantly reduced ventricular myocardial volume by 47%–58,485 ​± ​7525 ​μm^3^ (mean ​± ​SEM) compared to 124,177 ​± ​8660 ​μm^3^ in siblings (a pool of all other embryos with two or more wild type alleles) (p ​= ​0.002, Fig. 4A and B). Ventricular width was 22% lower in *mef2ca*^*+/*^^−^*;mef2cb*^*−/−*^ mutants (65 ​± ​6 ​μm) compared with siblings (87 ​± ​3 ​μm) (p ​= ​0.005, Fig. 4B), whereas ventricle length was unaltered (data not shown), implying that elongation was successful but a defect in ballooning was present. Trabecular length was not significantly different between *mef2ca*^*+/*^^−^*;mef2cb*^*−/−*^ mutants and siblings (Fig. 4B). In addition, measuring total ventricular wall volume of fixed 3 dpf *myl7:EGFP* fish (also stained for MyHC) showed that average ventricle volume of *mef2ca*^*+/*^^−^*;mef2cb*^*−/−*^ fish is significantly smaller (by 42%) than that of their siblings *mef2ca*^*+/+*^*;mef2cb*^*+/*^^*−*^ (Figs. S2D and E). At 5 dpf, *mef2ca*^*+/*^^−^*;mef2cb*^*−/−*^ ventricle had increased in volume but remained 35% smaller than siblings’ ventricle, 105,255 ​± ​13,443 ​μm^3^ compared with 163,226 ​± ​10.017 ​μm^3^ (p ​= ​0.013, Fig. 4A and B). The *mef2ca*^*+/*^^−^*;mef2cb*^*−/−*^ ventricle is also significantly smaller than that of *mef2cb*^*−/−*^ (161,167 ​± ​9913 ​μm^3^) (p ​= ​0.03, Fig. 4A and B). The other genotype possessing a single wild type allele, *mef2ca*^*−/−*^*;mef2cb*^*+/*^^−^, showed no defect in ventricular size for any parameter at either 3 or 5 dpf (Fig. 4A,C). Taken together, the data show that a single wild type allele of *mef2cb* is sufficient for achieving correct ventricular size by 3 dpf, whereas one wild type allele of *mef2ca* alone fails to support normal ventricle formation on a *mef2cb* mutant background.

### 3.4 CM addition in *mef2ca*^*+/*^^−^*;mef2cb*^*−/−*^ embryos is defective before 3 dpf, but normal thereafter

**Fig. 5:**
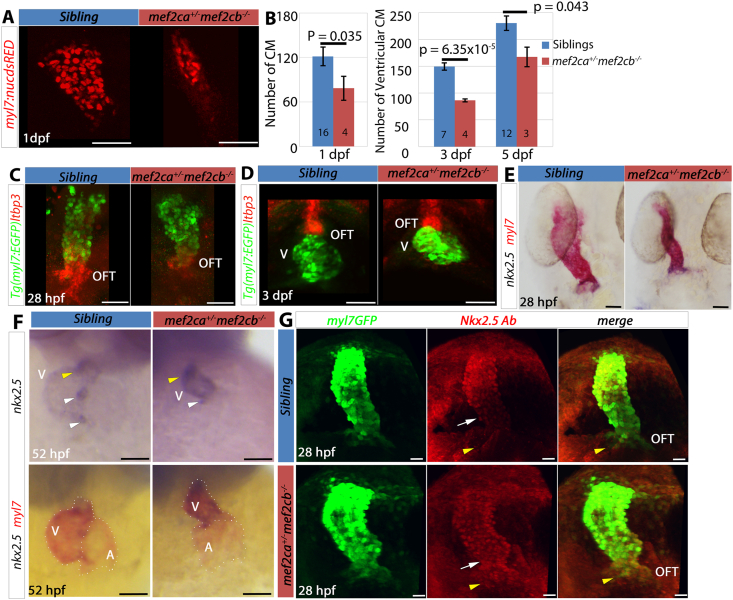
***Mef2ca***^**+/**^^−^**;*mef2cb***^**−/−**^**mutant embryos have reduced CM and changes in SHF gene expression. (A,B)** Embryos from a cross of *mef2ca*^*+/*^^−^*;mef2cb*^*+/*^^−^*;Tg(myl7:EGFP)* and *mef2cb*^*+/*^^−^*;Tg(myl7:EGFP);Tg(myl7:nucDsRed)* were fixed and immunostained with GFP (green) and RFP (red) antibodies at 1 dpf or imaged live at 3 and 5 dpf and CM nuclei counted. Confocal stacks are shown on red channel only at 1 dpf for clarity (A). Nuclei number of *mef2ca*^*+/*^^−^*;mef2cb*^*−/−*^ (red bars) was significantly lower than in siblings (blue bars) at all stages (Unpaired Student’s t-test, P-values indicated on graph). Values are mean ​± ​S.E.M. N shown on bars. **(C,D)** Embryos from a cross of *mef2ca*^*+/*^^−^*;mef2cb*^*+/*^^−^*;Tg(myl7:EGFP)* to *mef2cb*^*+/*^^−^ at 28 hpf (C) and 3 dpf (D) hybridised for *ltbp3* mRNA (Fast Red) and *myl7:EGFP* detected with anti-GFP antibody. Hearts of *mef2ca*^*+/*^^−^*;mef2cb*^*−/−*^ showed less differentiated myocardium (*myl7:EGFP*) co-expressing *ltbp3* mRNA and *ltbp3* levels outside the heart were also reduced (C), but by 3 dpf little difference was seen between genotypes (D). **(E,F)** In situ mRNA hybridisation for *nkx2.5* (blue) and *myl7* (red) in embryos from a cross of *mef2ca*^*+/*^^−^*;mef2cb*^*+/*^^−^ and *mef2cb*^*−/−*^ at 28 hpf (E, dorsal view) and 52 hpf (F, ventral view, same embryos shown in top panel for *nkx2.5* only and in bottom panel for *nkx2.5* and *myl7*). *nkx2*.5 ​mRNA was upregulated slightly at 28 hpf but was clearly upregulated throughout the ventricle and down in the atrium at 52 hpf, beyond the normal strong expression in AVC (white arrowhead) and the arterial pole (yellow arrowhead). Dotted line (bottom panel, F) marks chambers. **(G)** Confocal stack of immunodetection of Nkx2.5 and GFP (*myl7:EGFP*) of hearts of *mef2ca*^*+/*^^−^*;mef2cb*^*+/*^^−^ and sibling at 28 hpf in dorsal view showing Nkx2.5 upregulation in the ventricular region of the heart tube (white arrow) and in the arterial pole (yellow arrowhead). Scale bars ​= ​50 ​μm.

To test the hypothesis that reduced levels of Mef2c lead to a smaller ventricle by reduction in the number of CMs that differentiate, ventricular CMs were counted in mutant and sibling embryos/larvae at 1, 3 and 5 dpf. At 1 dpf, *mef2ca*^*+/*^^−^*;mef2cb*^*−/−*^ mutants had 78 ​± ​16 nuclei (n ​= ​4) in the whole heart tube compared to 121 ​± ​8 nuclei in siblings (n ​= ​16), a reduction of around 43 CMs (35%) compared to wild type (p ​= ​0.035, Fig. 5A and B). At 3 dpf, *mef2ca*^*+/*^^−^*;mef2cb*^*−/−*^ mutants may have gained a few nuclei, having 86 ​± ​3 ventricular nuclei (n ​= ​4), but numbers remained low compared with 149 ​± ​7 ventricular nuclei in siblings (n ​= ​7), such that the deficit in *mef2ca*^*+/*^^−^*;mef2cb*^*−/−*^ mutants had widened to ~63 (43%) fewer ventricular CMs (p ​= ​6 ​× ​10^−5^, Fig. 5B). In striking contrast, by 5 dpf *mef2ca*^*+/*^^−^*;mef2cb*^*−/−*^ mutants had gained many CM nuclei, reaching a total of 167 ​± ​18 (n ​= ​3) compared with siblings, which had 231 ​± ​13 nuclei (n ​= ​12; p ​= ​0.042), only a 28% deficit. Although mutants still lagged behind their siblings at 5 dpf, the deficit in ventricular CM number in *mef2ca*^*+/*^^−^*;mef2cb*^*−/−*^ larvae remained numerically similar to that at 3 dpf at around 64 CMs fewer than siblings. In both mutants and siblings the number of ventricular CMs increased by around 82 during the 3 to 5 dpf period, suggesting CM addition was normal in mutants over this period. Thus, the early defect in embryonic CM addition in *mef2ca*^*+/*^^−^*;mef2cb*^*−/−*^ mutants recovers to a normal pace in the larval heart.

### 3.5 *Mef2ca* and *mef2cb* function upstream of *ltbp3* and downstream of *nkx2.5* in CM precursors of the arterial pole

Previous work has shown that disruption of CM progenitor cells in the SHF leads to smaller ventricle size at 2–3 dpf (de Pater et al., 2009; Guner-Ataman et al., 2013; Witzel et al., 2017; Zhou et al., 2011). To determine if reduction of Mef2c activity also affected the progenitor pool, we used the SHF marker *ltbp3*. *Ltbp3,* which encodes a protein that modulates TGF-β signalling, marks CM progenitor cells at the OFT (Zhou et al., 2011). Ltbp3 is required to generate arterial pole CM progenitors that are added to the ventricle and OFT before 72 hpf and knockdown of *ltbp3* results in a smaller ventricle but normal atrium by 72 hpf (Zhou et al., 2011), reminiscent of the *mef2ca*^*+/*^^−^*;mef2cb*^*−/−*^ phenotype. Expression of *ltbp3* overlaps with *mef2cb* expression during heart tube stages and partially overlaps with *myl7* expression (Witzel et al., 2017; Zeng and Yelon, 2014; Zhou et al., 2011). Indeed, *mef2ca*^*+/*^^−^*;mef2cb*^*−/−*^ embryos had reduced *ltbp3* mRNA at 28 hpf, showing the progenitor pool is affected by reduction of Mef2cs (Fig. 5C), suggesting Mef2c regulates *ltbp3* mRNA levels. Double labelling with *myl7:EGFP* showed reduced *ltbp3* mRNA staining outside the heart tube. Moreover, there is also less overlap between *myl7* and *ltbp3* marked regions suggesting reduction in recently differentiated CMs (Fig. 5C), consistent with reduced CM number seen in the heart tube (Fig. 5A). By 3 dpf, no obvious reduction in *ltbp3* was detected near the heart OFT (Fig. 5D), a result that correlated with the recovery of CM addition to the arterial pole. Taken together, the data suggest that the early defect in CM addition to the ventricle in Mef2c-deficient embryos is at least partly due to Mef2c regulation of *ltbp3* expression in CM progenitors at the arterial pole of the heart tube.

As we show for Mef2c, *nkx2.5* (together with *nkx2.7*) functions upstream of *ltbp3* to drive cardiomyogenesis in the arterial pole SHF (Guner-Ataman et al., 2013; Targoff et al., 2013). Bioinformatic analysis of the *ltbp3* proximal promoter reveals multiple potential binding sites for Mef2c and one for Nkx2.5 (TRANSFAC, Fig. S4A). Nkx2.5 and Nkx2.7 were also shown to promote expression of *mef2cb* (Colombo et al., 2018). Comparative genome analysis reveals two evolutionary conserved regions (ECR) in the *mef2cb* locus that had 70–75% identity with mouse and human, both containing Nkx2.5 binding sites (Fig. S4B). It is possible, therefore, that Nkx2.5/Nkx2.7 regulate *ltbp3* either directly or through Mef2c. Alternatively, Mef2c could regulate *ltbp3* via effects on *nkx2.5* and/or *nkx2.7* genes, because we have previously shown that *nkx2.*5 ​mRNA is diminished in non-differentiating CMs in *mef2ca;mef2cb* double mutants (Hinits et al., 2012). To distinguish between these possibilities, we examined *nkx2.*5 ​mRNA in *mef2ca*^*+/*^^−^*;mef2cb*^*−/−*^ mutants. At 28 hpf, despite reduced *myl7* mRNA accumulation in *mef2ca*^*+/*^^−^*;mef2cb*^*−/−*^ mutants, we could not detect changes in *nkx2.5* expression compared with other genotypes with more Mef2c (Fig. 5E). However, by 52 hpf *nkx2.*5 ​mRNA appeared to be upregulated in the ventricles of *mef2ca*^*+/*^^−^*;mef2cb*^*−/−*^ embryos (Fig. 5F; 5/7 genotyped embryos; 0/12 controls showed similar upregulation). Raised *nkx2.*5 ​mRNA levels were detected only in newly differentiated CMs at the arterial pole and the AVC in controls, and no *nkx2.5* expression was observed outside the OFT (Fig. 5F). Using anti-Nkx2.5 antibody, we found that the upregulation in the ventricular CMs is detected already at 28 hpf (Fig. 5G), and it is noted in precursors in the arterial pole that are positive for Nkx2.5 but are negative for myl7:EGFP or show only weak myl7:EGFP (Fig. 5G). Thus, depleted Mef2c seem to upregulate Nkx2.5 levels in myocardial precursors. At 2–3 dpf, no *nkx2.*5 ​mRNA or protein is detected outside the heart (Fig. 5F, S3A). Similar results were obtained using a *Tg(nkx2.5:ZsYellow)* line, in which neither mRNA for *nkx2.5* nor ZsYellow was observed in precursor cells outside the OFT at 72 hpf (Paffett-Lugassy et al., 2017). We therefore tested whether the Nkx2.5 transgene GFP^+^ cells near the OFT (Fig. 2E), which indicate previous Nkx2.5 expression, were altered in Mef2c deficient embryos. To do so we crossed *mef2ca*^*+/*^^−^*;mef2cb*^*+/*^^−^;*TgBAC(nkx2.5:GAL4FF)*^*hu9579*^*;Tg(UAS:h2az2a-GFP)*^*hu685*1^*)* to *mef2cb*^*−/−*^. No *mef2ca*^*+/*^^−^ fish expressed the reporter, suggesting close linkage of the integration site of one of the transgenes to the *mef2ca* locus on chromosome 10 and thus preventing further analysis. In contrast, *mef2cb*^*−/−*^ embryos did not have significantly more *GFP*^+^ ​precursors outside the OFT (Figs. S3B and C). Thus, in summary, Mef2cs act either in parallel with, or downstream of, *nkx2.5* and both genes act upstream of *ltbp3* in driving the addition and differentiation of ventricular CMs from the arterial pole. In addition, Nkx2.5 is upregulated (or fails to be downregulated) in ventricular CMs as well as in their precursors when Mef2cs are depleted.

The recovery of CM addition after 3 dpf may reflect a change in the dependency of CM differentiation from Mef2ca/Mef2cb to other Mef2s. (Fig. S5). *Mef2ca* and *mef2cb* are expressed early in the heart tube, and *mef2cb* mRNA is abundant in the cells differentiating and entering the OFT region until about 2 dpf but, by 3 dpf, *mef2ca* and *mef2cb* mRNAs were downregulated near the heart (Colombo et al., 2018; Hinits et al., 2012; Lazic and Scott, 2011) and Figs. S5A–C). At 3 dpf, the dominant Mef2 mRNA detected was that from *mef2aa* that expresses strongly in the heart, including in the differentiating precursors at the arterial pole (Figs. S5A–C).

### 3.6 Adult *mef2ca*^*+/*^^−^*;mef2cb*^*−/−*^ are viable but smaller than siblings

**Fig. 6:**
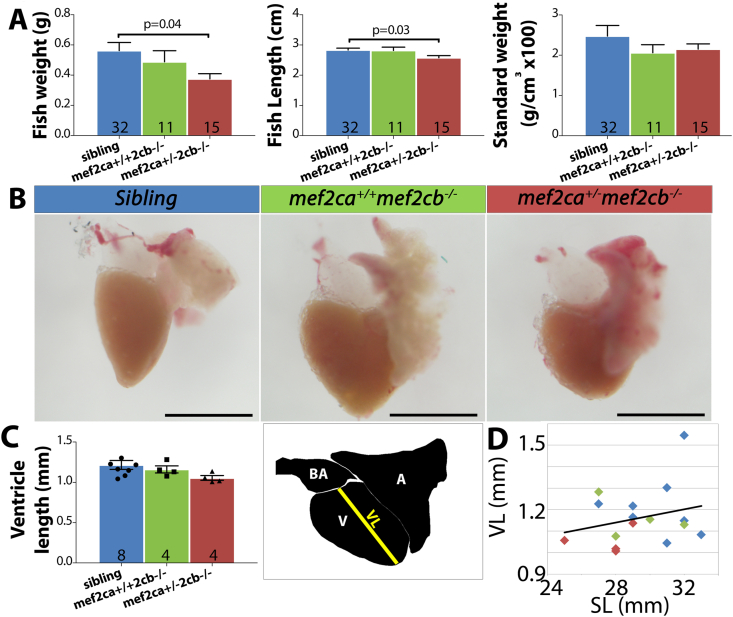
***Mef2ca***^***+/***^^−^***mef2cb***^***−/−***^**adults are viable but small. (A)** Weight and standard length (SL) of genotyped three to four month old adult fish were measured and standard weight (K) calculated. *Mef2ca*^*+/*^^−^*;mef2cb*^*−/−*^ mutant adults had a significantly lower BW and SL compared with their siblings. Graphs show mean ​± ​S.E.M. for pooled data from 2 lays. N for each group shown on bars. One-way ANOVA was performed followed by Dunnett test for multiple comparison. **(B**–**D)***Mef2ca*^*+/*^^−^*;mef2cb*^*−/−*^, *mef2cb*^*−/−*^ and siblings at 4 months of age were measured and their heart dissected and imaged (B). Ventricle length (VL) was measured from images from the centre of the bulbous to the apex of the heart (diagram in C). Graph shows mean ​± ​S.E.M. N for each group shown on bars. VL was plotted vs. SL (D). Note that points for *mef2ca*^*+/*^^−^*;mef2cb*^*−/−*^ mutants fall below the linear regression line. Scale bars ​= ​1 ​mm.

Despite the apparent return to a normal pace of CM addition from 3 to 5 dpf, ventricle size at 5 dpf is still significantly reduced in *mef2ca*^*+/*^^−^*;mef2cb*^*−/−*^ mutants (Fig. 4). To investigate whether this defect affected further development and survival of *mef2ca*^*+/*^^−^*;mef2cb*^*−/−*^ mutants into adulthood, two separate crosses producing *mef2ca*^*+/*^^−^*;mef2cb*^*−/−*^ embryos were reared to adulthood. *mef2ca*^*+/*^^−^*;mef2cb*^*−/−*^ mutants were viable and fertile as adults. Genotyped crosses that were analysed at 3–4 months of age yielded the expected numbers of progeny of each genotype, with the exception of total absence of double mutants. In a cross between *mef2ca*^*+/*^^−^*;mef2cb*^*+/*^^−^ and *mef2ca*^*+/+*^*;mef2cb*^*−/−*^, 25/82 (30%) were found to be *mef2ca*^*+/*^^−^*;mef2cb*^*−/−*^ (χ^2^ ​= ​0.251) and in a cross between *mef2ca*^*+/*^^−^*;mef2cb*^*+/*^^−^ and *mef2ca*^*+/+*^*;mef2cb*^*+/*^^−^, 6/51 (12.5%) were found to be *mef2ca*^*+/*^^−^*;mef2cb*^*−/−*^ (χ^2^ ​= ​0.874). We measured two lays of adult fish around 3–4 months for length and weight. Interestingly, *mef2ca*^*+/*^^−^*;mef2cb*^*−/−*^ mutants are significantly smaller in both weight and length compared with their siblings (Fig. 6A), although standard weight (SW), which compensates for length change (SW ​= ​weight (g) x 100/[length (cm)]^3^) was not significantly different (Fig. 6A). To test whether the reduction of Mef2c had affected the heart, we dissected hearts from 4 month old fish and measured ventricular length (VL), which did not differ significantly between *mef2ca*^*+/*^^−^*;mef2cb*^*−/−*^ mutant and sibling hearts (Fig. 6B and C). In juvenile and adult zebrafish, VL is correlated to standard length (SL) and VL/SL averages around 4.3% (varies between 2.7 and 7.8%) throughout life (Singleman and Holtzman, 2012). Despite all *mef2ca*^*+/*^^−^*;mef2cb*^*−/−*^ mutants remaining below the trend line (Fig. 6D), all Mef2c genotypes tested had a VL/SL figure within the normal range between 3.3% and 4.8% (Fig. 6D). Thus, despite the embryonic CM deficit and size difference, *mef2ca*^*+/*^^−^*;mef2cb*^*−/−*^ mutants are viable and have functional hearts of normal size as adults.

## 4 Discussion

The findings of this work demonstrate three major points. Firstly, we show that the developing heart grows beyond 3 dpf by significant increase in ventricular myocardial volume, primarily due to trabeculation and outward expansion. The growth is achieved by both proliferation of existing CMs and addition of CMs from a pool of precursors from 3 to 5 dpf, along with cellular hypertrophy of individual CMs. Secondly, we show that normal ventricular growth prior to 3 dpf requires a threshold level of Mef2c, and this threshold diminishes after 3 dpf. Thirdly, adult *mef2ca*^*+/*^^−^*;mef2cb*^*−/−*^ fish with significantly smaller heart at 3 dpf survive to adulthood but are smaller than their siblings.

### 4.1 Modes of growth of the ventricular myocardium after cardiac looping

We investigated cellular mechanisms (modes) contributing to the cardiac growth observed between 3 and 5 dpf. The myocardial wall of the heart tube could balloon outwards during chamber formation (Auman et al., 2007; Christoffels et al., 2000; Moorman and Christoffels, 2003), leading to outward growth accompanied by myocardial thinning. Alternatively, the myocardial wall could thicken, either by hypertrophy or cell division. In addition, trabeculation, by repositioning CMs, gives inward growth into the lumen of the ventricle (Liu et al., 2010; Peshkovsky et al., 2011; Staudt et al., 2014), and could thus thin the myocardial wall between trabeculae. A combination of these growth modes must be occurring between 3 and 5 dpf. Our data suggest around 5% of existing CMs in both chambers pass through S phase between 4 and 5 dpf, consistent with work using a FUCCI (fluorescent ubiquitylation-based cell cycle indicator) system (Choi et al., 2013). Choi et al. observed 4–5% CMs in S/G2/M phases between 3 and 5 dpf in the whole heart. These findings contrast with another study using a slightly different FUCCI system, which showed around 14% of CMs expressing *Tg(myl7:mVenus-gmnn)* and 9% of CMs labelled by 6 ​h of EdU at 5 dpf, and suggested proliferation is the main contributor to CM number increase between 3 and 5 dpf (Uribe et al., 2018). Like Uribe et al. we also examined M-phase CMs with PH3 and observed low numbers of 0 or 1 PH3^+^ CMs per embryo. Low CM proliferation rates were also found by using PH3 and BrdU at 24–48 hpf (de Pater et al., 2009). Assuming that among the approximately 200 CMs present at 3 dpf, one (0.5%) is in an M-phase lasting 15 ​min (a typical value in many eukaryotic cells) and 10–28 (5–14%) are in S/G2/M, then S/G2/M must last between 3 and 9 ​h, a range congruent with the 7–8 ​h estimate from time lapse imaging by Uribe et al. (2018). Labelling of 5–10% of CMs after 6 ​h in EdU, as observed by Uribe et al. thus suggests that ~30% of CMs undergo mitosis per day, which would lead to a larger increase in CMs (138) than that observed. In another experimental series, Uribe et al. observed 15% of CMs labelled by EdU in 24 ​h, again predicting a larger increase in CMs (64) than we observed. In contrast, we only observed about 5% of CMs (i.e. 10) in S phase over 24 ​h of EdU labelling, suggesting that no more than 10 ​cells undergo mitosis per day, insufficient to account for the 50 CMs added between 3 and 5 dpf. Definitive resolution of this issue will require sustained high-resolution time-lapse imaging to track and quantify CM increase. Nevertheless, our results suggest that proliferation cannot account for all the growth observed.

We find that CM progenitor cells are present in a continued CM progenitor field at the arterial pole beyond 2 dpf and contribute to myocardial growth. CMs also undergo hypertorphy, such that the myocardial wall thickens, despite ballooning and trabeculation. Thus, the modes of growth occurring during the well-investigated 1–2 dpf period (de Pater et al., 2009; Gessert and Kühl, 2009; Hami et al., 2011; Lazic and Scott, 2011; Zhou et al., 2011) persist until 5 dpf. However, by 5 dpf, either the extra-cardiac progenitor pool is depleted or changes by losing expression of its known markers. Interestingly, our Kaede experiments show a few myocardial regions with newly-made Kaede-Green only CMs. Although these areas could theoretically reflect mosaic clonal activation of a previously silent UAS:Kaede reporter leading to patches of green-only cells, we believe this unlikely for three reasons. First, transgene mosaicism is generally thought to be the result of position effect variegation, which is not expected to alter synchronously and reproducibly in local groups of cells. Second, we do not observe an obvious increase in overall CM labelling, as would be expected if the reporter gradually became active as the heart developed. Third, we found no evidence for differential turnover of kaede in these cells. We prefer the simpler interpretation that green-only cells are newly-differentiated CMs. In the case of the arterial pole area this may reflect the addition of CMs from an external pool, as suggested also by the concentration of newly-differentiated CMs in this region at 3.5 dpf in the developmental timing assay. Other areas like the AVC and the apex of the ventricle could be hotspots for proliferation as proliferation would dilute the converted protein overtime, and support the clonal distribution of cardiomyocytes seen in young juveniles (Gupta and Poss, 2012). Interestingly, the apex of the ventricle is where in similar developmental stages in the mouse (E12 and later) some SHF myocardial cells contribute to the ventricular septum as seen by the *Mef2c*-AHF-Cre fate map (Verzi et al., 2005).

The main possible source for cells added to the arterial pole after 3 dpf is the group of cells outside the OFT that have previously expressed *nkx2.5*, *isl 1* and/or *isl2a/2b*, which at 3 dpf are only marked by retained fluorescent protein expression of transgenes. Lineage tracing of nearby cells after 3 dpf is required to determine the extent and source of the external pool of cells yielding new CMs in larval life.

### 4.2 Mef2cs are required for CM addition

Redundant roles have been suggested for different Mef2 factors across several tissues in various organisms (Arnold et al., 2007; Assali et al., 2019; Desjardins and Naya, 2016; Hinits and Hughes, 2007; Hinits et al., 2012; Lin et al., 1997; Potthoff et al., 2007; Sacilotto et al., 2016). Due to genome duplication in teleosts, there are two Mef2c factors in the zebrafish. Our results show a clear difference in the requirement for Mef2ca and Mef2cb function. While normal levels of Mef2ca can be sufficient for normal heart development (this work and (Hinits et al., 2012)) a single wild type allele of *mef2ca* is insufficient to support myocardial growth in the absence of Mef2cb. However, a single allele of *mef2cb* in *mef2ca*^*−/−*^*;mef2cb*^*+/*^^−^ mutants permits ventricular growth indistinguishable from wild type. *Mef2ca* and *mef2cb* are both expressed early in each heart field and in the heart tube, but *mef2cb* mRNA alone is abundant in cells differentiating and entering the OFT region around 48 hpf. Taken together, these findings suggest that the function of both proteins is similar and the difference in functional requirement stems from their different expression patterns. In mice there is only a single *Mef2c* gene, but early development of the heart tube occurs in *Mef2c* null mutants, possibly aided by a compensatory mechanism in which *Mef2b* mRNA is upregulated 7-fold (Lin et al., 1997). In contrast, later SHF development is severely affected by lack of Mef2c (Lin et al., 1997; Vong et al., 2005). Interestingly, although we observe a similar reduction in cardiomyogenesis from 1 to 3 dpf in mutants with low Mef2c activity, our data show that *mef2ca*^*+/*^^−^*;mef2cb*^*−/−*^ mutants recover and add the same number of CMs between 3 and 5 dpf as their siblings. A major difference from mice is that zebrafish entirely lacking a heart can develop well for many days, whereas mice that are unable to build a functional four-chambered heart die from anoxia (Bi et al., 1999; Lin et al., 1997, 1998; Materna et al., 2019). Our finding of an early time window when SHF contribution to the myocardium is more sensitive to loss of Mef2c activity than either earlier or later cardiomyogenesis thus appears similar to the murine data available. We hypothesise the *mef2aa* activity may rescue myocardial growth post-embryonically in the zebrafish.

### 4.3 Effects of embryonic defects in later life

We observe reduced size of adult *mef2ca*^*+/*^^−^*;mef2cb*^*−/−*^ fish. Could the substantial defects in myocardium of 3 dpf *mef2ca*^*+/*^^−^;*mef2cb*^*−/−*^ mutants account for the adult size reduction? We think it possible but unlikely that the loss of just fifty early CMs has a lifelong effect, at least under aquarium conditions. As *mef2ca* and *mef2cb* are expressed in zebrafish skeletal muscle (Hinits and Hughes, 2007; Yogev et al., 2013), one attractive possibility is that the smaller body size is due to reduced growth in skeletal muscle. Mice with a skeletal muscle conditional knockout for *Mef2c* have reduced body weight from around postnatal day 10 into adulthood along with fibre type disproportion and altered glucose metabolism (Anderson et al., 2015). Alternatively, the small body size in *mef2ca*^*+/*^^−^;*mef2cb*^*−/−*^ mutant fish arise from craniofacial defects as *Mef2c* in mouse and *mef2ca* in fish were found to be essential for craniofacial development (DeLaurier et al., 2014; Miller et al., 2007; Nichols et al., 2016; Verzi et al., 2007). Non-lethal jaw defects in zebrafish, seen also for some *mef2ca* allelic combinations (Miller et al., 2007) can lead to insufficient feeding and small body weight, as we found in scleraxis mutants (Kague et al., 2019). Finally, as fish and mouse Mef2cs express in tissues such as CNS, bone, smooth muscle and other cell types (Anderson et al., 2004; Arnold et al., 2007; Barbosa et al., 2008; Hammond and Udvadia, 2010), it is possible later defects unrelated to the muscle explain the growth phenotype.

Interestingly, Mef2 genes are upregulated upon cardiac injury in zebrafish (Wang and Poss, 2016), which could relate to their ability to regenerate the myocardium. Encouragingly, our data argue that low residual Mef2c function in our single allele mutant combinations appears sufficient to support CM proliferation in the 3–5 dpf period. Recently, two studies have identified two individual *MEF2C* mutations in patients with SHF-related congenital heart disease (CHD) (Lu et al., 2018; Qiao et al., 2017), highlighting the importance of correct levels and function of MEF2C in human heart. Whether physiological regeneration and exercise tests on *mef2ca*^*+/*^^−^;*mef2cb*^*−/−*^ adult fish would reveal cardiac defects remains to be determined.

### 4.4 Mef2c place in the cardiac transcriptional cascade

How does Mef2c activity fit into the hierarchy of SHF cardiomyogenesis? The downregulation of *ltbp3* and up-regulation of *nkx2.5* observed in *mef2ca*^*+/*^^−^;*mef2cb*^*−/−*^ mutants at 28 hpf precedes the defects in ventricular growth at 2 and 3 dpf. Small ventricles have been reported in various zebrafish mutants and deficits such as *nkx2.5*^*−/−*^ (Targoff et al., 2013) and *ltbp3*^*MO*^ (Zhou et al., 2011). The reduction of the *ltbp3* mRNA in *mef2ca*^*+/*^^−^;*mef2cb*^*−/−*^ mutants implies a reduction in progenitor pool size at 1 dpf. As Ltbp3 is involved in CM progenitor proliferation (Nevis et al., 2013; Zhou et al., 2011), reduction in *ltbp3* expression could further decrease CM progenitors. *Nkx2.5*, on the other hand, is implicated in specification of CMs and is also required for *ltbp3* expression (Colombo et al., 2018; Guner-Ataman et al., 2013; Strate et al., 2015; Targoff et al., 2013). Our data show that even in the presence of raised *nkx2.*5 ​mRNA and protein in precursor cells, *ltbp3* is not activated and thus Mef2c activity is required in parallel with, or mediates, Nkx2.5 activity on *ltbp3* expression. This understanding, together with results from *nkx2.5/nkx2.7* mutants showing that the Nkx2 factors are required for *mef2cb* expression (Colombo et al., 2018) places *mef2cb* between *nkx2.5/nkx2.7* and *ltbp3* in the genetic cascade of the SHF in zebrafish.

We previously showed that in double mutant fish completely lacking Mef2c activity, specification of FHF CM precursors occurred correctly prior to heart tube formation and the bilateral heart fields were indistinguishable from sibling embryos. However, in the absence of Mef2c protein, differentiation markers were reduced and specified cells were lost. Expression of *nkx2.5* was diminished when progenitors failed to differentiate (Hinits et al., 2012). It is also possible that Mef2c binds to Nkx2.5 regulatory elements to allow Nkx2.5 recruitment and autoregulation as shown for mice and other vertebrates (Clark et al., 2013). Here, we find that when Mef2c activity is low, but differentiation occurs, ventricular and atrial CMs respond differently; ventricular CMs upregulate *nkx2.5* expression, which may reflect a function of Nkx2.5 in maintaining ventricular identity (George et al., 2015; Targoff et al., 2013). Low levels of Mef2c can therefore not just prevent many CMs from differentiating and incorporating into the ventricle but the ones that do differentiate and beat may fail to mature through the effects of reduction of Mef2c on Nkx2.5 levels.

## Declaration of competing interest

All authors have no competing financial interest.
