# Supplementary material for: Mef2c factors are required for early but not late addition of cardiomyocytes to the ventricle
Source: Dev Biol. 2021 Feb;470:95–107. doi: 10.1016/j.ydbio.2020.11.008 (PMC7819464; doi:10.1016/j.ydbio.2020.11.008)
Supplement: Multimedia component 1 [file mmc1.pdf]

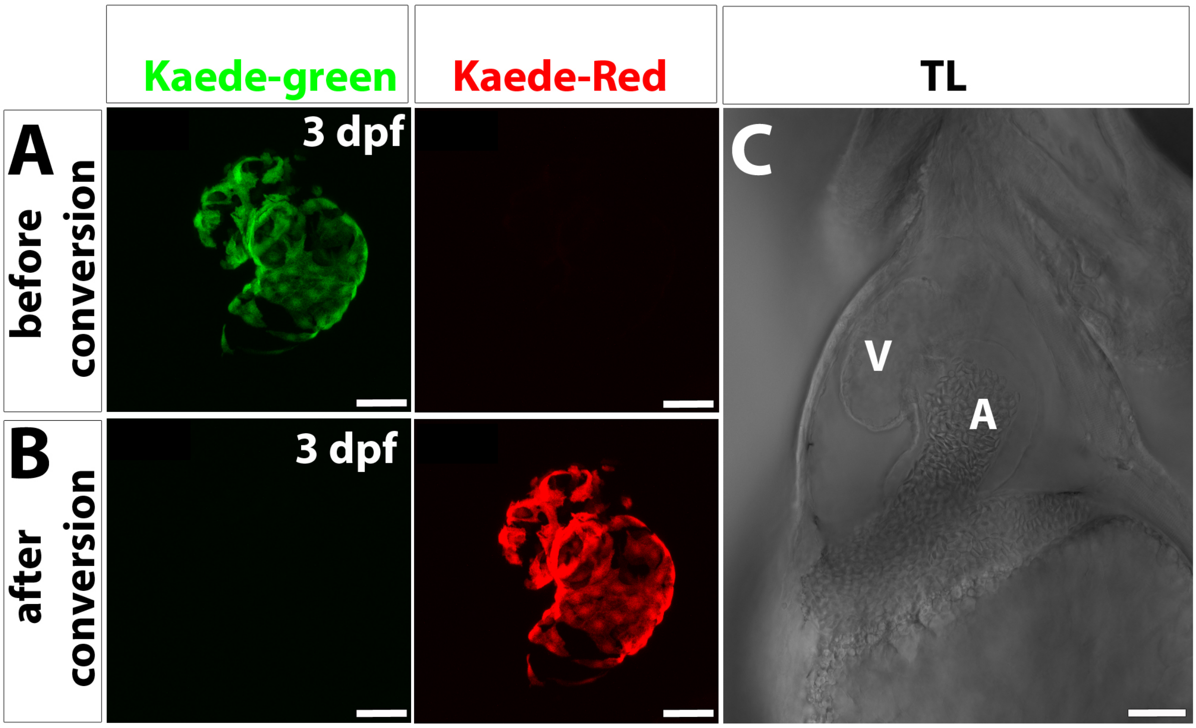

Supplementary Figure S1. Kaede conversion at 3 dpf.

Confocal stacks of hearts of 3 dpf embryos carrying *Tg(myl7:gal4FF)*^{hu6531} *and Tg(UAS:Kaede)*^{rk8} before (A) and after (B) Kaede-green was converted to Kaede-red. Transmitted light of the scanned area is shown in C. A, atrium, V, ventricle. Scale bars = 50 μm.

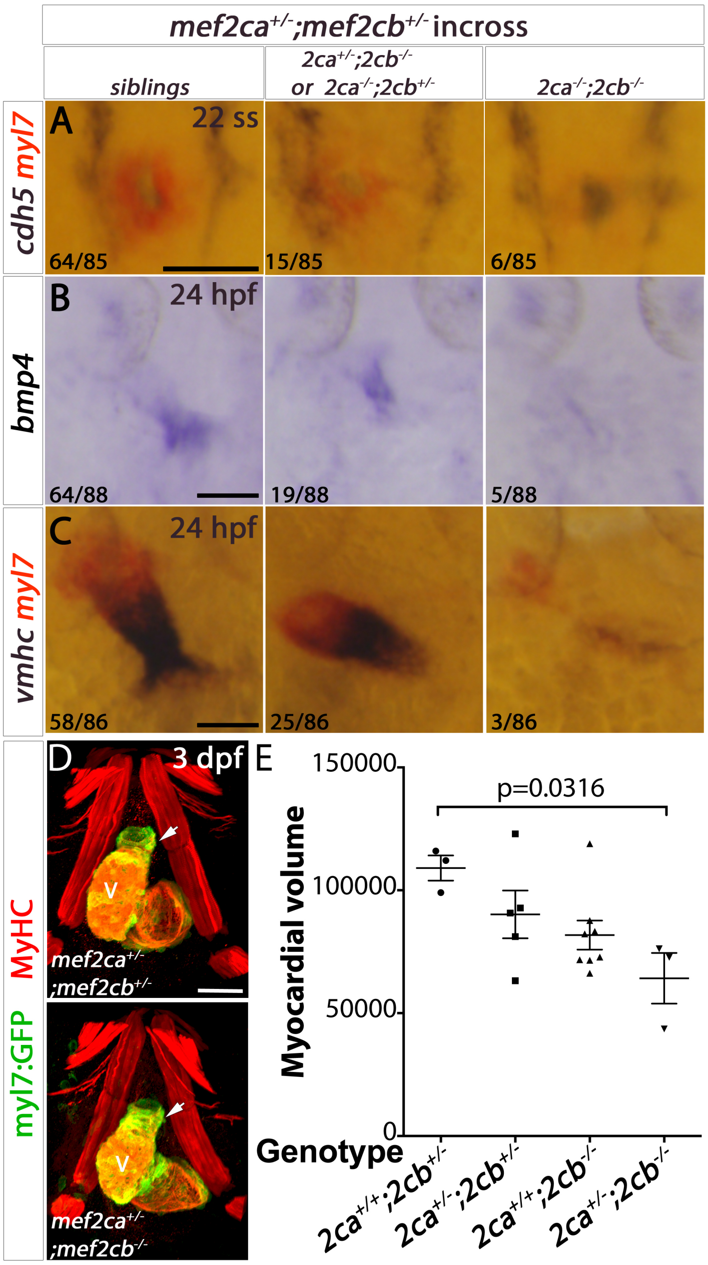

Supplementary Figure S2. Incrosses of *mef2ca*$^{+/-}$*;mef2cb*$^{+/-}$ give rise to an array of heart phenotypes. (A-C). In situ mRNA hybridisation for *cdh5* and *myl7* (A) at 22 ss, *bmp4* (B), *vmhc* and *myl7* (C) at 24 hpf in hearts of zebrafish embryos shown in a dorsal view, anterior to top. Heavy loss of myocardial differentiation markers in the heart is seen in a fraction likely to be *mef2ca*$^{-/-}$*;mef2cb*$^{-/-}$ mutants (right panels), whereas a larger fraction show a milder reduction (middle panels) compared with normal looking hearts (left panels). Numbers of embryos (ungenotyped) are indicated on panels. **(D,E)** Confocal stacks of hearts from 3 dpf embryos from a cross between *Tg(myl7:EGFP)*; *mef2ca*$^{+/-}$*;mef2cb*$^{+/-}$ and *mef2cb*$^{-/-}$ stained for GFP antibody (green) and MyHC (red) showing a smaller, mis-shaped ventricle in *mef2ca*$^{+/-}$*;mef2cb*$^{-/-}$, with undeveloped OFT (white arrow). Graph (E) shows average ventricle volumes across the various genotypes. *mef2ca*$^{+/-}$*;mef2cb*$^{-/-}$ volume is significantly lower than that mef2ca$^{+/+}$;mef2cb$^{+/-}$. Data shown as mean +/- SEM. Statistics: one-way ANOVA followed by Tukey's multiple comparisons test. Scale bars = 100 µm, except in D = 50 µm.

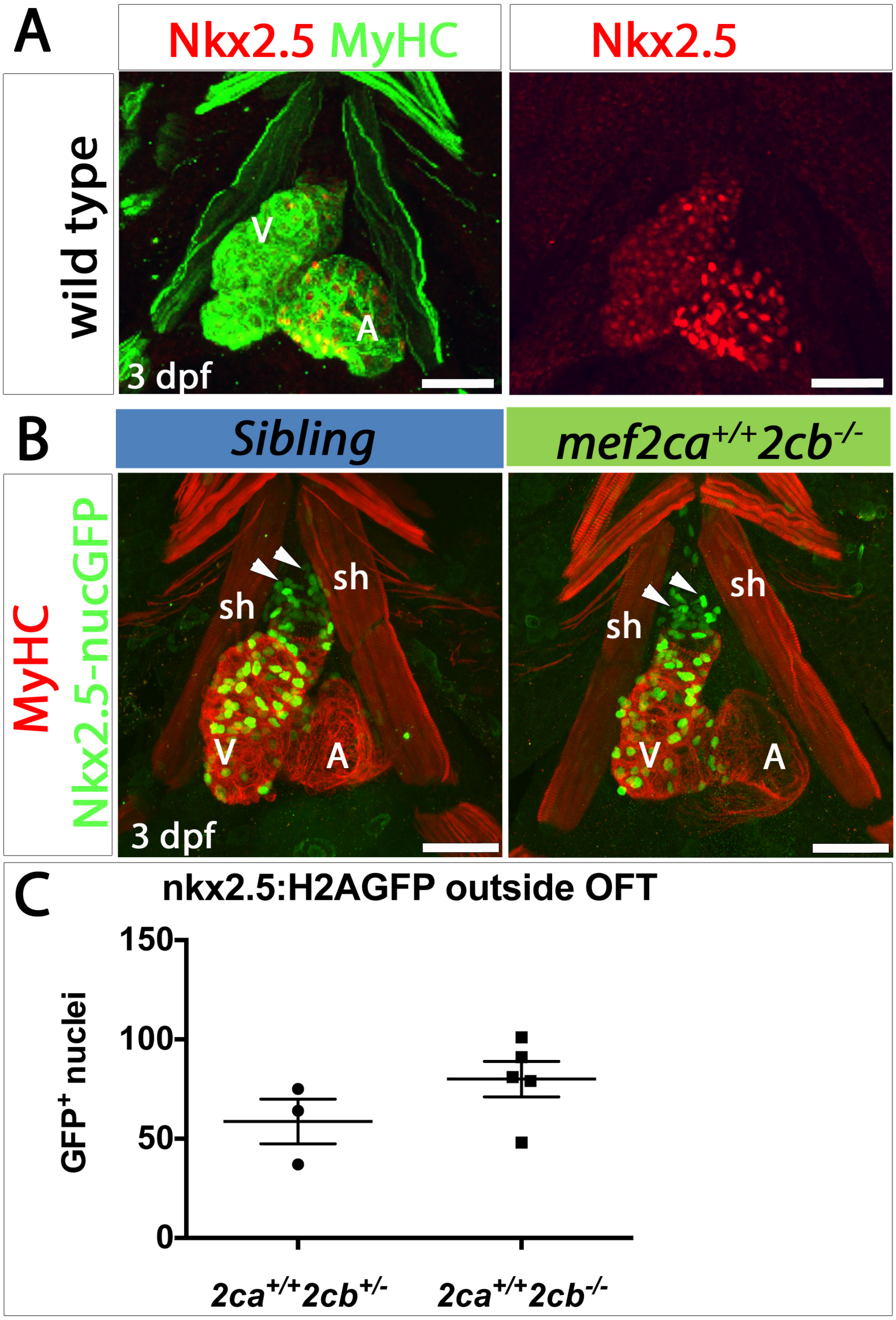

Supplementary Figure S3. Nkx2.5 protein and reporter expression. (A) Confocal stacks of 3 dpf wild type embryos stained for Nkx2.5 antibody (red) and MyHC (A4.1025, green). **(B,C).** Confocal stack of embryos from a cross of *mef2ca*$^{+/-}$*;mef2cb*$^{+/-}$*; Tg(nkx2.5BACgalFF); Tg(UAS:h2a-gfp)* and *mef2cb*$^{-/-}$ genotyped and immunostained for GFP (green), and MyHC (A4.1025, red). GFP^{+} nuclei (nkx2.5$^{+,}$ arrowheads) outside the differentiated ventricle, near the OFT were counted using the sternohyoides muscle inner edge as a border, and shown in C. Scale bars = 50 µm. A, atrium, V, ventricle, sternohyoides (sh).

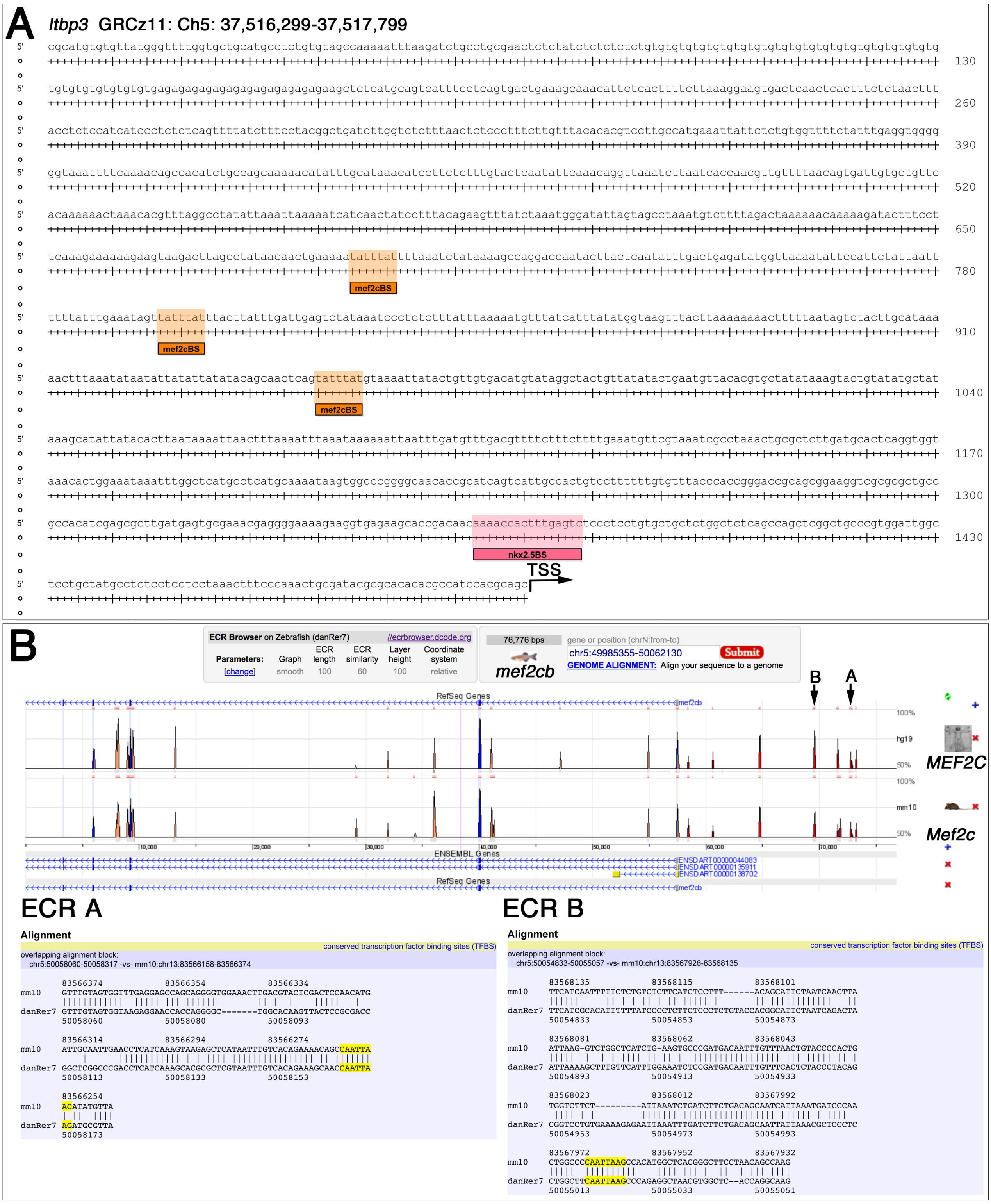

Supplementary Figure S4. Transcription factor analysis in *ltbp3* and *mef2cb* sequences. A. Sequence of 1.5 Kb of proximal promoter of *ltbp3* is shown with Mef2c and Nkx2.5 binding sites according to analysis using TRANSFAC (Wingender, 2008) **B.** Comparative analysis of *mef2cb* locus using Evolutionary Conserved Region (ECR) browser (http://www.dcode.org) (Ovcharenko et al., 2004) and rVISTA 2.0 (Loots and Ovcharenko, 2004) found two ECRs, labelled A and B, that contain a conserved potential Nkx2.5 binding site, highlighted in an alignment with the mouse *Mef2c* and human *MEF2C* sequences. Zebrafish ECR A has 71% identity with both human and mouse, whereas ECR B has 75% and 70% identity with human and mouse, respectively. Colour codes for peaks: blue: coding exons, yellow: UTRs, salmon: intronic regions, red: intergenic regions, pink line: ECRs.

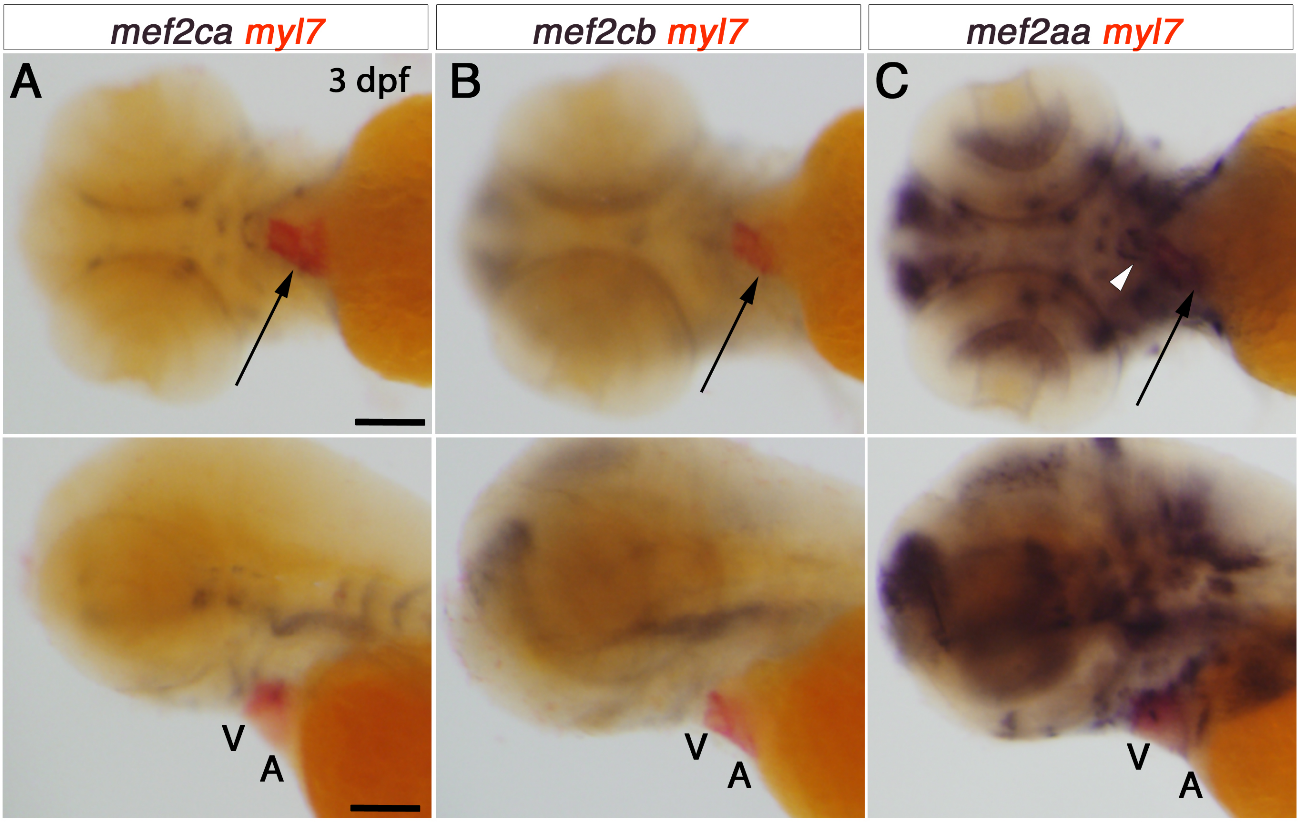

Supplementary Figure S5. Expression of mef2s at 3 dpf. (A-C). Wholemount in situ mRNA hybridisation for myl7 (in red) and *mef2ca (A),* mef2cb (B) and mef2aa (C) at 72 hpf, shown in ventral view (top panels) and lateral view (bottom panels). Only *mef2aa* mRNA is strongly expressed in the heart at this time, especially the ventricle (arrows) and the OFT (white arrowhead). A, atrium, V, ventricle. Scale bars = 100 µm.

Supplementary References

Loots, G.G., Ovcharenko, I., 2004. rVISTA 2.0: evolutionary analysis of transcription factor binding sites. Nucleic Acids Res 32, W217-221.

Ovcharenko, I., Nobrega, M.A., Loots, G.G., Stubbs, L., 2004. ECR Browser: a tool for visualizing and accessing data from comparisons of multiple vertebrate genomes. Nucleic Acids Res 32, W280-286.

Wingender, E., 2008. The TRANSFAC project as an example of framework technology that supports the analysis of genomic regulation. Brief Bioinform 9, 326-332.
